# Progestin-Only contraceptive and clinical complications in women with sickle cell disease: a prospective study

**DOI:** 10.61622/rbgo/2026rbgo57

**Published:** 2026-09-23

**Authors:** Flávia Anchielle Carvalho da Silva, Evelyne Nascimento Pedrosa, Manuela Freire Hazin-Costa, Ariani Impieri Souza

**Affiliations:** 1 Instituto de Medicina Integral Prof Fernando Figueira Recife PE Brazil Instituto de Medicina Integral Prof. Fernando Figueira, Recife, PE, Brazil.; 2 Faculdade Pernambucana de Saúde Recife PE Brazil Faculdade Pernambucana de Saúde, Recife, PE, Brazil.

**Keywords:** Contraception, Hormonal contraception, Anemia sickle cell, Sickle cell disease, Progestins, Contraceptive agents, Pain Headache

## Abstract

**Objective:**

To evaluate the influence of progestin-only contraceptives use on the occurrence of clinical complications in women with sickle cell disease.

**Methods:**

A prospective, non-randomized, comparative study was conducted involving 54 women with sickle cell disease between March 2018 and August 2020. Were excluded the women that were pregnant or wanted to become pregnant, were over 40 years old, and were not sexually active at the time of recruitment. Following contraceptive counseling, participants were monitored at 3, 6, and 12 months. They were classified into three groups: progestogen only, combined hormonal, and non-hormonal contraceptives users. Clinical complications were assessed throughout the follow-up. Data analysis was performed using logistic regression models for repeated binary outcomes, applying the generalized estimating equation method with robust variance. The value of *p*<0.05 was considered statistically significant.

**Results:**

All participants experienced pain crises during their lives. No significant differences were observed between the groups regarding the frequency of pain crises. However, the progestogen only group presented a significantly lower frequency of headaches at 3, 6, and 12 months (p=0.012, p=0.016, and p=0.021, respectively) compared to the combined hormonal group.

**Conclusion:**

Contraceptives containing progestogen only were not associated with a reduction in pain episodes in women with sickle cell disease but were associated with a lower frequency of headaches compared to combined hormonal contraception.

## Introduction

Over the years, the survival of people with Sickle Cell Disease (SCD) has increased worldwide, and women reaching reproductive age face pregnancy challenges. Physiological changes during pregnancy, such as dilutional anemia, hypercoagulability, and immunosuppression, worsen the clinical parameters of SCD and increase morbimortality in this population, even in developed countries.^(1,2)^

Given the increased risk of morbidity and the lack of effective therapies to improve pregnancy outcomes, contraceptive counseling is one of the strategies used to prevent unwanted pregnancies and their deleterious consequences.^(3,4)^ Despite this, women with SCD continue to become pregnant without planning, even though they have received information about the risk of pregnancy and contraceptive counseling.^(4,5)^

According to the World Health Organization (WHO) eligibility criteria, revised in 2025,^(6)^ women with Sickle Cell Disease (SCD) can use any contraceptive method (CM). However, robust scientific evidence for an ideal CM for these women is lacking.^(3)^ Therefore, each patient should be evaluated individually, as many women in this population have comorbidities that may contraindicate the use of certain CM.^(4)^

Theoretically, both SCD and the use of combined hormonal contraceptives (CHC) may increase the risk of thromboembolic events, but contraceptive safety among women with SCD remains limited.^(7)^ In contrast, a prospective multicenter study published in 2015 of 1,257 women with SCD showed a 4-fold increase in the incidence of ischemic stroke in women with SCD using CHC.^(8)^

In 2024, the CDC revised its eligibility criteria for contraceptive methods and raised the risk of using CHC for women with SCD to risk level 4 but advises considering individual risks and disease severity,^(9)^ while the WHO maintains a risk level of 2 for women with SCD using CHC in 2025.^(6)^

Furthermore, the WHO emphasized the use of depo-medroxyprogesterone acetate (DMPA) to reduce vaso-occlusive crisis (VOC) in women with SCD.^(10)^ Given the absence of a clear consensus on the optimal CM for women with SCD, this study aimed to evaluate the influence of progestogen only contraceptives (POC) on the occurrence of VOC and other clinical complications in this population.

## Methods

This prospective, non-randomized, comparative study was conducted at a reference hospital for women's healthcare in Recife, Pernambuco, Brazil, from 2018 to 2020. The sample size calculation was based on a study that showed a reduction in the frequency of VOC with the use of POC compared to the use of CHC.^(5)^ In the present study, we set out to find a 50% reduction in the frequency of VOC with the use of POC compared to other CM, adopting a power of 90% and a significance level of 5% (two-tailed). Thus, the sample size was estimated to be 30 women in each group (POC, non-hormonal methods, and CHC). Considering a 20% loss, the sample size increased to 36 women in each group.

During the recruitment period (March 2018–August 2019), 116 women were contacted and invited to participate but only 54 were included. Women were excluded if they were pregnant or wanted to become pregnant, were over 40 years old, had the HbSC genotype, and were not sexually active at the time of recruitment.

Initially, biological, sociodemographic, reproductive, and clinical data were collected. The CM for women to choose were then presented, considering the WHO eligibility criteria.^(6)^ The women received the chosen CM and were followed up for one year. The CM available at the service were: DMPA, intrauterine device (IUD) with levonorgestrel, copper IUD, progestogen-only pill, combined pill (ethinyl estradiol-0.02 mg/ levonorgestrel-0.10 mg), male condom, and definitive methods (tubal ligation or vasectomy). All CM were free of charge.

Women returned to the clinic or were contacted by telephone at 3, 6, and 12 months after starting CM use. During the follow-up, the primary endpoint regarding the frequency of VOC was monitored, along with the secondary endpoints: frequency of headache, other clinical complications, and occurrence of pregnancy.

Data collection was reviewed and entered a database in Excel©, version 2007, and statistical analyses were performed using *Stata* software, version 12.1. Tables of absolute and relative frequencies were created for categorical variables, and measures of central tendency and dispersion were created for numerical variables. To correlate the time of use of each CM with the frequency of VOC and headaches in the year before entering the study. The Spearman's correlation coefficient was used.

All eligible participants were included in the analysis, considering three groups: POC, CHC, and non-hormonal method. The comparison of the percentage frequencies of VOC and headache among the three contraceptive methods and over the three visits (3, 6, and 12 months) was performed by fitting a logistic regression model for repeated binary outcomes using the generalized estimating equation method with robust variance.^(11)^ The value of *p*<0.05 was considered statistically significant.

This study has been conducted in accordance with the principles set forth in the Helsinki Declaration and was approved by the Institutional Research Ethics Committee on December 13^th^, 2017, under CAAE:70666317.8.0000.5201 and approval number:2.349.314. All participants provided written informed consent prior to inclusion in the study

## Results

During the follow-up period, 54 women were classified into three groups: 27 in the POC group, 6 in the CHC group, and 21 in the non-hormonal method group. The distribution of SCD genotypes was as follows: 52 (96.3%) had HbSS, and two (3.7%) had S-β-thalassemia. The mean age was 27.7(±5.2) years old. Most participants were black (88.9%), had nine years or more of schooling (87.0%), and had no income on their own (87.0%). Most patients were already pregnant (83.3%), and among these, 44 reported complications during a prior pregnancy (97.8%). The mean number of living children is 1.3(±0.5). When asked about the CM used before inclusion in the study, 35.2% used male condoms, 29.6% used CHC, 13.0% used POC, and 13.0% used tubal ligation. The median time of CHC use was nine months (IIQ:4-24), POC use was 44 months (IIQ:10-48) and non-hormonal methods use was 24 months (IIQ:6-108). Regarding clinical history, all women had episodes of VOC, and 45(83.3%) had at least one episode in the previous year. Thromboembolic events were reported in 12 women (22.2 %). Headaches or migraines were reported by 51(94.4%) patients, 21 of whom reported at least seven episodes in the past year (Tables 1, 2 and 3).

**Table 1:** Sociodemographic, biological and background data

Variables	n(%)
Genotype	
HbSS	52(96.3)
S-βtalassemia	2(3.7)
Age (years): Mean+SD	27.7+5.2
Mixed/Black race	48(88.9)
Schooling ≥ 9 years	47(87.0)
No income	47(87.0)
Prior pregnancy	45(83.3)
Previous numbers of pregnancy: Mean+SD	1.6±0.9
Number of living children: Mean+SD	1.3±0.5
Complication in previous pregnancy	44(97.8)
Previous CM* used at the time prior to the study	
Condom	19(35.2)
Combined hormonal contraceptive (CHC)	16(29.6)
Progestogen-only pill	7(13.0)
Tubal ligation	7(13.0)
Frequency of VOC** in the last year	
1-2	26(48.1)
≥3	19(35.2)
Did not present last year	9(16.7)
Past thromboembolic event	12(22.2)
Headache/migraine	51(94.4)
Frequency of headaches in the year prior the study≥ 7	21(41.2)

* Previous contraceptive methos

** Vaso-occlusive crisis

**Table 2:** Multiple comparisons among contraceptive methods M1, M2 and M3

Follow-up time	M2 vs M1 difference: M2–M1	M3 vs M1 difference: M3–M1	M3 vs M2 difference: M3–M2
3 months	8.6(p =0.394)	9.4(p =0.479)	0.8(p =0.952)
6 months	9.8(p =0.381)	10.7(p =0.466)	0.9(p =0.852)
12 months	7.6(p =0.400)	8.3(p =0.480)	0.8(p =0.952)

M1: Progestogen only contraceptive (POC); M2: Non-hormonal method; M3: Combined Hormonal Contraceptive (CHC)

**Table 3:** Multiple comparisons among contraceptive methods M1, M2 and M3

Follow-up time	M2 vs M1 difference: M2–M1	M3 vs M1 difference: M3–M1	M3 vs M2 difference:M3–M2
3 months	8.9(*p*=0.464)	35.8(*p*=0.012)	26.9(*p*=0.097)
6 months	7.9(*p*=0.453)	28.0(*p*=0.016)	20.1(*p*=0.128)
12 months	8.0(*p*=0.473)	28.7(*p*=0.021)	20.6(*p*=0.086)

M1: Progestogen only contraceptive (POC); M2: Non-hormonal method; M3: Combined Hormonal Contraceptive (CHC)

Progestogen only contraceptives showed the lowest percentage of VOC over the follow-up period (3, 6, and 12 months), while the non-hormonal and CHC showed similar percentages over time. Despite this, the differences between the POC and the other two groups were not statistically significant (Figure 1).

**Figure 1:**
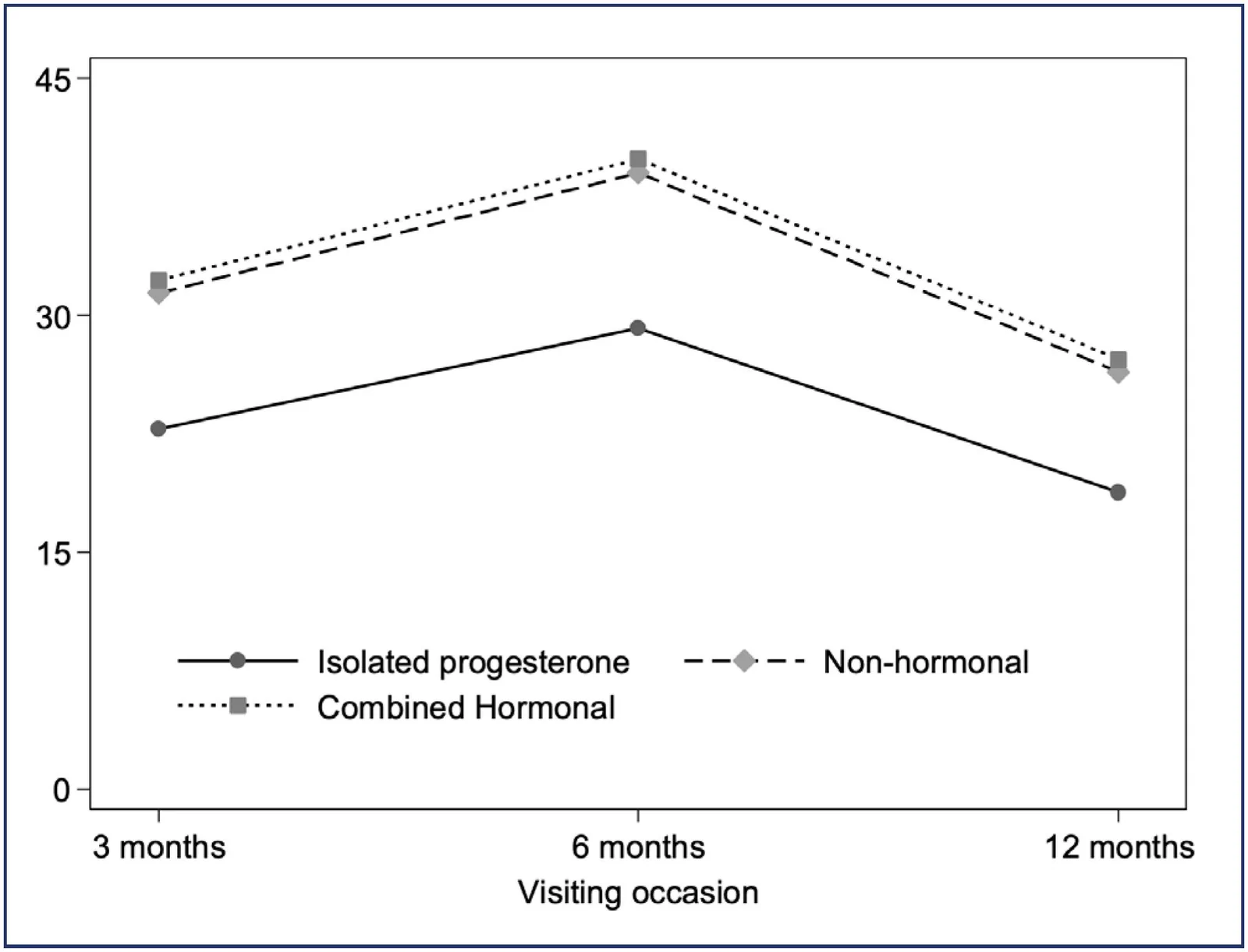
Frequency of vaso-occlusive crisis (VOC), according to contraceptive method over time

Regarding the relationship between headache episodes and CM use, POC presented the lowest percentage over time, whereas CHC presented the highest percentage. Statistically significant differences were observed in the number of events between POC and CHC over the three time points: 3(*p*=0.012), 6(*p*=0.016), and 12(*p*=0.021) months (Figure 2).

**Figure 2:**
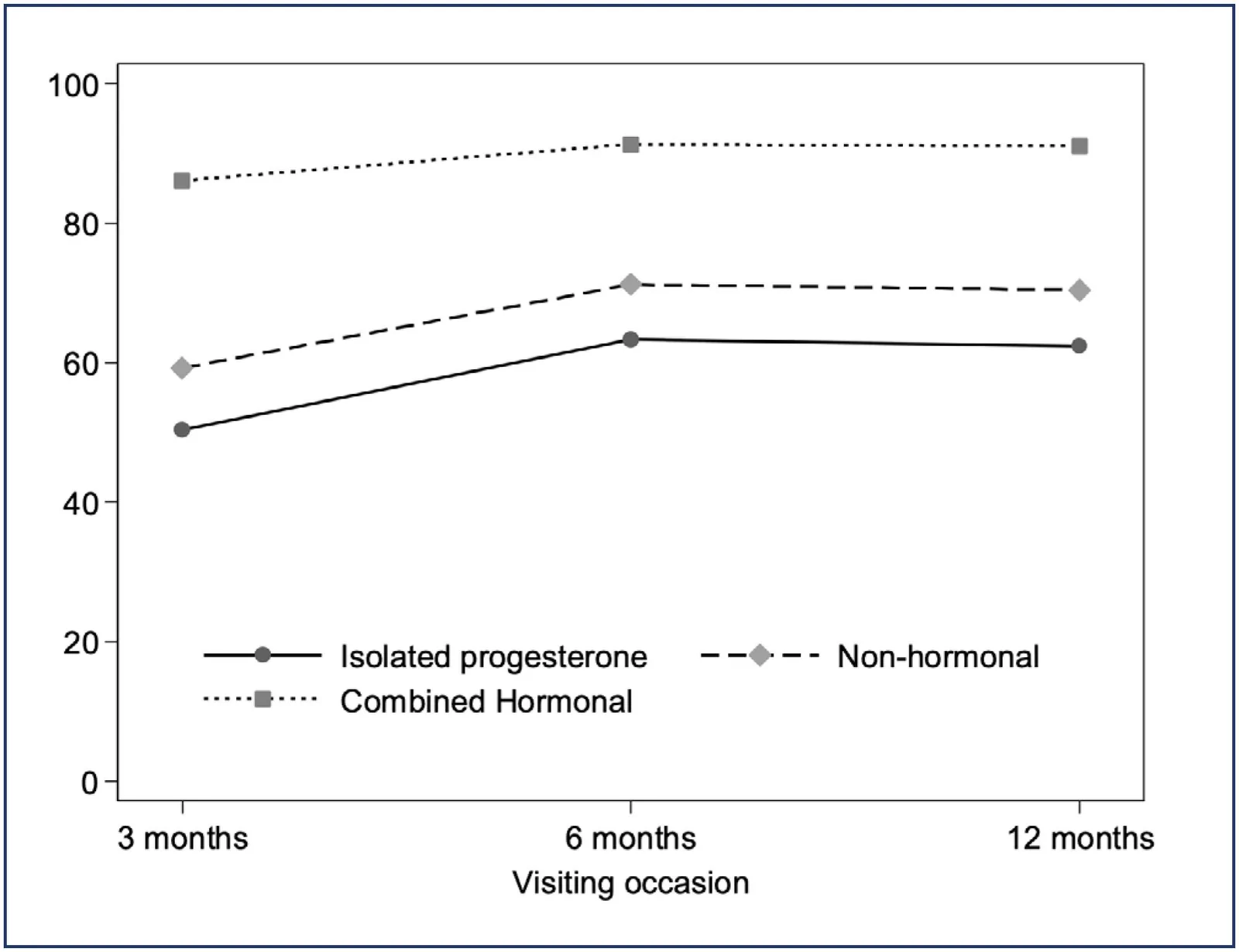
Frequency of headache, according to contraceptive method over time

No pregnancies occurred during the follow-up period. There were two cases of thromboembolic events in the non-hormonal group. One patient with a history of stroke had a deep vein thrombosis episode after six months of follow-up, and another had a stroke episode after 12 months of follow-up.

## Discussion

The women in this sample were mostly young, black race, of the HbSS genotype, with more than nine years of schooling, no income of their own, and who had already been pregnant. Among the contraceptive methods used before enrolling in the study, most participants used condoms. All women reported having had a VOC.

The women's mean age in this study did not differ from that of others related to family planning and reproductive profile and either women with SCD or the general population.^(4,5,12)^ The fertility rate found in this sample was lower than the average of the Brazilian population (1.3 versus 1.7 live children/woman),^(13)^ which is related to the fact that almost all women reported complications during their prior pregnancy. This reinforces the need for contraceptive counseling in this population to reduce unwanted pregnancies.^(2,14)^

Most women were already using some CM at the time of entry into the study, with a predominance of non-hormonal ones, similar to that found in the literature on women with SCD.^(4,5)^ In contrast, CHC is most commonly used by the general population.^(15)^ One explanation for this difference lies in the risk of thromboembolic events associated with the use of combined hormonal contraceptives (CHC), as well as in people SCD.^(7,9)^

When the time of use of CM before entering the study was evaluated, those with POC were associated with the longest time of use, followed by non-hormonal, and finally, CHC. This higher adherence of POC may be explained by the fact that they offer greater convenience (quarterly injectable and hormonal IUD) and have few side effects.^(4,15)^

Although evidence is limited, the CDC recently raised the risk of thrombosis with the use of DMPA for women with SCD,^(7,9)^ but studies have shown fewer pain crises with the use of POC, including DMPA.^(16)^

VOC was the primary clinical complication for women in this study, and most reported having at least one episode of VOC in the year before the study entry, which is consistent with what is found in the literature.^(17)^

A higher prevalence of headaches in women with SCD has also been reported; however, the underlying mechanism is not yet understood.^(18)^ In this study, the percentage of patients with recurrent headaches was higher than that found in population-based studies, as well as those evaluating people with SCD.^(17,19,20)^ Other factors that may explain the high incidence of headaches in this study were female sex, low socioeconomic status, and HbSS, which is the most severe SCD genotype.^(18,20)^

In this study, POC use was not associated with a reduction in VOC frequency over the follow-up period when compared with other CM. The lack of this association was similar to studies that evaluate several types of progestin presentation together (pill, injectable, and IUD).^(3)^ This suggests that the reduction in VOC frequency could depend on the type, dose, and route of progesterone administration. However, studies with a larger number of patients with the same progesterone presentation are required to elucidate its association with VOC frequency.

When evaluating headache frequency over time, the frequency of POC was lower than that of CHC. A systematic review showed that desogestrel alone seems promising for reducing migraine frequency in women.^(21)^ However, data regarding the type of CM and headache frequency in women with SCD are scarce. Another systematic review described the results of a clinical trial of a subdermal implant (containing only etonogestrel, a progestin) which showed a reduction in headache over time in women with SCD.^(3)^

Finally, it is worthwhile to remember that the proportion of thromboembolic events in young women aged 20-40 years old in the general population is between 5-10 per 100,000/year.^(22)^ In the case of women with SCD, this is explained by the fact that the condition increases the risk of these events due to endothelial lesions caused directly by sickle cells, as well as by the chronic inflammatory reaction that promotes endothelial damage.^(23,24)^ This fact highlights the need for careful evaluation of these women when choosing a contraceptive method.^(25)^

Despite limitations related to small sample size, particularly for comparisons involving CHC users, progestin-only contraceptives were not associated with a significant reduction in VOC, however, a lower frequency of headaches was observed compared with CHC, suggesting a potential beneficial effect that should be further investigated in larger studies.

Furthermore, SCD is a health condition that leads to complications in pregnancy, making it essential that women with SCD who do not wish to become pregnant are properly advised to use safe contraceptive methods and that studies continue to be conducted to improve the evidence and demonstrate which methods are most suitable for these women.

## Conclusion

Contraceptives containing progestogen only were not associated with a reduction in pain episodes in women with sickle cell disease but were associated with a lower frequency of headaches compared to combined hormonal contraception.

## Data Availability

The research data are described in the article presented.
